# Bio-Based Epoxy Shape-Memory Thermosets from Triglycidyl Phloroglucinol

**DOI:** 10.3390/polym12030542

**Published:** 2020-03-02

**Authors:** David Santiago, Dailyn Guzmán, Francesc Ferrando, Àngels Serra, Silvia De la Flor

**Affiliations:** 1 Eurecat—Chemical Technologies Unit, c/Marcel·lí Domingo 2, 43007 Tarragona, Spain; dailyn.guzman@eurecat.org; 2 Department of Mechanical Engineering, Universitat Rovira i Virgili, Av. Països Catalans 26, 43007 Tarragona, Spain; f.ferrando@urv.cat (F.F.); silvia.delaflor@urv.cat (S.D.l.F.); 3 Department of Analytical and Organic Chemistry, University Rovira i Virgili, c/Marcel·lí Domingo 1, 43007 Tarragona, Spain; angels.serra@urv.cat

**Keywords:** shape-memory polymers, bio-polymers, epoxy, renewable resources

## Abstract

A series of bio-based epoxy shape-memory thermosetting polymers were synthesized starting from a triglycidyl phloroglucinol (3EPOPh) and trimethylolpropane triglycidyl ether (TPTE) as epoxy monomers and a polyetheramine (JEF) as crosslinking agent. The evolution of the curing process was studied by differential scanning calorimetry (DSC) and the materials obtained were characterized by means of DSC, thermogravimetric analysis (TGA), dynamic mechanical analysis (DMA), stress-strain tests, and microindentation. Shape-memory properties were evaluated under free and totally constrained conditions. All results were compared with an industrial epoxy thermoset prepared from standard diglycidyl ether of Bisphenol A (DGEBA). Results revealed that materials prepared from 3EPOPh were more reactive and showed a tighter network with higher crosslinking density and glass transition temperatures than the prepared from DGEBA. The partial substitution of 3EPOPh by TPTE as epoxy comonomer caused an increase in the molecular mobility of the materials but without worsening the thermal stability. The shape-memory polymers (SMPs) prepared from 3EPOPh showed good mechanical properties as well as an excellent shape-memory performance. They showed almost complete shape-recovery and shape-fixation, fast shape-recovery rates, and recovery stress up to 7 MPa. The results obtained in this study allow us to conclude that the triglycidyl phloroglucinol derivative of eugenol is a safe and environmentally friendly alternative to DGEBA for preparing thermosetting shape-memory polymers.

## 1. Introduction

Shape-memory polymers (SMPs) are materials that can change their shape upon application of an external stimulus. They can be deformed and fixed in a new or temporary shape, which will be stable until the application of the stimulus [1]. This shape-changing is called the shape-memory effect (SME) and it is usually heat-triggered, although it can also be induced by means of other sources, such as light, electricity, or magnetic fields [2,3,4].

SMPs are usually classified according to their chemical nature (thermoplastic or thermosets) and transition temperature (glass or melting transition temperature) [5]. Thermosetting SMPs have been extensively studied due to their good mechanical properties, high recovery and fixity ratios, high tensile modulus below the transition temperature, and excellent rubber elasticity above the transition temperature [5,6]. Among them, epoxy-based shape-memory thermosets are probably the most used thermosetting SMPs [7,8]. 

Epoxy resins are widely used in many applications, such as coatings, adhesives, or matrices in composites because of their chemical resistance, thermal stability, and good mechanical properties [9]. The most common starting compound in the production of epoxy resins is bisphenol A, a petroleum-based chemical. Aromatic compounds, such as bisphenol A, are widely used in organic materials as they confer stability, toughness, and good thermal and mechanical properties to epoxy thermosets. However, an excessive exposure to bisphenol A may lead to serious damage to health [10]. For that reason, many studies were carried out to obtain epoxy thermosets from renewable sources. The most common natural sources to produce epoxy compounds are vegetable oils [11,12,13] and cardanol [14,15], but they can also be obtained from lignin [16], rosin [17], tannin [18], and carbohydrates [19]. In addition, in recent years, some breakthrough work has been published. Qi et al. studied the preparation of epoxy resin precursors from biomass and magnolol [20,21].

Several works can be found in the literature about bio-based epoxy SMPs. Li et al. [22] synthesized bio-based diamine and epoxy monomer derived from isosorbide via the microwave assistant thiol-ene coupling reaction in an aqueous medium. The cured resin showed good shape-memory properties: near 100% shape-recovery ratio, 97% shape-fixity ratio, and a fast shape-recovery speed. Tsujimoto et al. [23] synthesized bio-based epoxy materials from epoxidized soybean oil (ESO) and epoxidized linseed oil (ELO) with 4-methylhexahydrophthalic anhydride (MHPA) as a curing agent. The materials showed shape-memory properties although the authors only evaluated them in qualitative way. Li et al. [17] synthesized a bio-based shape memory epoxy resin from rosin. The rosin-based epoxy showed better thermal, mechanical, and shape-memory properties than its petroleum-based counterpart.

Our group has a wide expertise in the synthesis of epoxy monomers from natural resources. Previous studies with triglycidyl eugenol derivative, tetraglycidyl bis-eugenol derivative [24,25,26] and, more recently, with triglycidyl phloroglucinol [27], evidenced that these phenolic compounds are a good renewable alternative to commercial DGEBA because the rigidity of the aromatic structure leads to excellent thermal and mechanical properties. 

Phloroglucinol, with three phenolic groups in its compact structure, is one of the major components present in Ecklonia Cava (brown algae) but it can also be obtained by a synthetic route from benzene [28]. The most important application of this compound is in pharmaceutics because it has excellent antispasmodic properties [29]. This aromatic compound could also be used as feedstock for the preparation of epoxy thermosets due to its great biocompatibility and good chemical characteristics. Several authors reported the preparation of thermosets starting from triepoxi phloroglucinol resin, and the results obtained demonstrated that these types of material are promising alternatives for DGEBA-based thermosetting polymers [30,31,32].

The main objective of this work is to obtain epoxy-based shape-memory thermosetting polymers starting from the bio-based triglycidyl phloroglucinol monomer (3EPOPh) and to demonstrate that it can be a good alternative to classical DGEBA-based thermosetting SMPs. In this sense, different formulations were prepared with 3EPOPh and different proportions of trimethylolpropane triglycidyl ether as epoxy monomers with stoichiometric proportions of a polyetheramine as crosslinking agent. Thermal and mechanical properties were evaluated by means of differential scanning calorimetry (DSC), dynamic mechanical analysis (DMA), stress-strain experiments and microindentation. Shape-memory properties were evaluated under free and constrained conditions.

## 2. Materials and Methods

### 2.1. Materials

**Figure 1:**
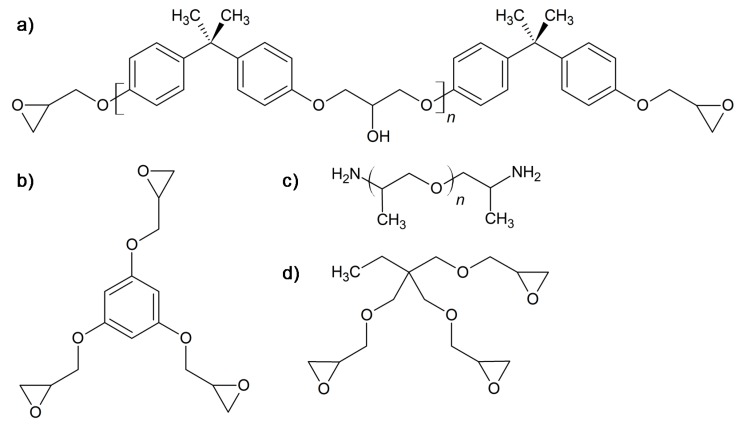
Chemical structure of DGEBA (**a**), triglycidyl phloroglucinol (3EPOPh) (**b**), Jeffamine^®^ D-400 (**c**), and trimethylolpropane triglycidyl ether (**d**).

Phloroglucinol, benzyl triethylammonium chloride (TEBAC) and epichlorohydrin (EPC) were purchased from Sigma-Aldrich (Sant Louis, MI, USA) and were used without further purification. Inorganic salts and bases were purchased from Scharlab (Barcelona, Spain). Chloroform, hexane, methylene chloride, and ethyl acetate were purchased from Carlo Erba (Milan, Italy) and were purified by standard procedures. DGEBA (Araldite GY 240) with a weight per epoxy equivalent of 182 g/mol and polyetheramine Jeffamine^®^ D-400 (JEF) (430 g/mol, 115 g/eq) were purchased from Huntsman (Chocolate Bayou, TX, USA). Trimethylolpropane triglycidyl ether (TPTE) (101 g/eq) was purchased from Sigma Aldrich (Sant Louis, MI, USA). Chemical structures of the starting compounds used in the preparation of the thermosets are depicted in Figure 1.

### 2.2. Synthesis of Triglycidyl Phlroglucinol Derivative

The synthesis of EPOPh (Figure 1b) was performed as follows: 10.49 g (0.06 mol) of phloroglucinol, 116.85 g (1.26 mol) of epichlorohydrin, and 2.60 g (0.01 mmol) of benzyltriethylammonium chloride (TEBAC) were stirred in a 500 mL flask at 100 °C for 4 h. The mixture was cooled down to 30 °C and then 90 mL of an aqueous solution of 20% NaOH and 2.60 g of TEBAC were added and maintained under stirring for 90 min. After that, 60 mL of ethyl acetate was added to the mixture for dilution. The phases were separated and the organic layer was washed twice with water and dried with magnesium sulfate. The solvent and excess epichlorohydrin were eliminated in a rotary evaporator at 60 °C. The resin obtained was purified by silica-gel chromatography using ethyl acetate/hexane 6/4 as eluent. The monomer obtained, is a white powder with 60% (10.58 g) yield. Mp: 50–55 °C. ^1^H NMR (CDCl_3_, δ in ppm): 6.1 s (Ar, 3H), 4.2 dd (-CH_2_-O-, 3H), 3.99 dd (-CH_2_-O-, 3H), 3.3 m (CH epoxy ring, 3H), 2.9 m (CH_2_ epoxy ring, 3H), and 2.7 m (CH_2_ epoxy ring, 3H). Furthermore, ^13^C NMR (CDCl_3_, δ in ppm): 160.2, 94.8, 68.8, 50.0, and 44.5. FT-IR (ATR): 3064, 3006, 2924, 2872, 2837, 1592, 1446, 1427, 1342, 1248, 1166, 1151, 1129, 1059, 984, 904, 858, 831 cm^−1^.

### 2.3. Preparation of Curing Mixtures

Four formulations were prepared with the compositions detailed in Table 1. Formulations were prepared by mixing the compounds in stoichiometric ratios of epoxy/NH_2_ groups and were homogenized by hand stirring using a spatula. The theoretical crosslinking density was calculated in two different ways. The first method is assuming that all the amine groups turn intro crosslinks (*υ_c_*); and the second method is by using the rubber elasticity equation (Equation (1)).

(1) $$E_{r}=3\cdot R\cdot T_{r}\cdot d\cdot \upsilon_{e}$$

where *E_r_* is the rubbery modulus, *R* is the universal constant gas (8.314 J/K·mol), *T_r_* is the temperature corresponding to *E_r_* (*T_g_* + 50) *d* is the mass density (assumed to be 1000 kg/m^3^ for the sake of simplicity), and *υ_e_* is the crosslink density. Both methods show very similar results, except in the case of formulation 3EPOPh-JEF, which shows higher crosslinking density when calculated with the theory of rubber elasticity equation. This discrepancy might be caused by topological restrictions such as loops or dangling.

**Figure 2:**
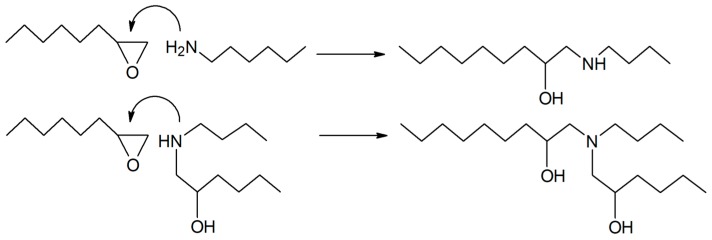
Simplified scheme of the epoxy-amine condensation mechanism.

All samples were cured for 2 h at 80 °C and 4 h at 160 °C and then polished with sandpaper to obtain the desired final dimensions. The molecular mechanism of the epoxy-amine condensation is shown in Figure 2.

### 2.4. Thermal Characterization

The study of the curing process was performed by differential scanning calorimetry (DSC) in a Mettler DSC3+ 700/970 (Columbus, OH, USA) calorimeter calibrated using an indium standard (heat flow calibration) and an indium-lead-zinc standard (temperature calibration). A flow of N_2_ at 100 mL/min was used and the weight of the samples for the analysis was ~10 mg. The curing process was studied by DSC in the non-isothermal mode at 10 °C/min from 20 °C to 275 °C. The glass transition temperatures (*T_g_*s) of the samples once cured were determined in dynamic scans at 20 °C/min, from 50 °C to 150 °C. 

The thermal stability of cured samples was studied by thermogravimetric analysis (TGA) using a Mettler TGA/SDTA 851e (Columbus, OH, USA) thermobalance. All experiments were performed under inert atmosphere (N_2_ at 100 mL/min). Pieces of the cured samples with an approximate mass of 8 mg were degraded between 30 °C and 600 °C at a heating rate of 10 °C/min.

### 2.5. Thermomechanical and Mechanical Characterization

Thermomechanical properties were measured using the TA Instruments DMA Q800 (New Castle, DE, USA) analyzer equipped with a 3-point bending clamp. Prismatic rectangular samples of about 30 mm × 6 mm × 2 mm were analyzed at 1 Hz, 0.1% strain and a heating rate of 3 °C/min from 30 °C to 150 °C. The glass transition temperature (*T_g_*) was determined from the maximum of the peak of tan δ. The values of storage modulus *E*′ below and above glass transition were evaluated. The onset of glass transition temperature *T_g_*^*E*′^ was determined as the peak of the loss modulus *E*″ curve.

Young’s modulus was determined under flexural conditions at room temperature, with the same clamp and geometry samples, applying a force ramp at constant load rate of 1 N/min, from 0.005 N to 1.5 N. Stress-strain tests were performed using the DMA Q800 equipped with a film-tension clamp in force-controlled mode. Dog-bone samples of about 20 mm × 1.5 mm × 0.5 mm were analyzed at *T_room_* and *T_g_*^*E*′^ at a force rate of 1 N/min. Microindentation hardness was measured with a Wilson Wolpert 401 MAV (Aachen, Germany) device following the ASTM E384-16 standard procedure. For each material, at least 15 determinations were made with a confidence level of 95%. The Vickers hardness number (HV) was calculated from the following equation [33]:

(2) $$HV=\frac{1.8544\cdot F}{d^{2}}$$

where, *F* is the load applied to the indenter in kgf (0.025 kgf) and *d* is the arithmetic mean of the length of the two diagonals of the surface area of the indentation measured after load removal in mm.

### 2.6. Shape-Memory Properties Characterization

**Figure 3:**
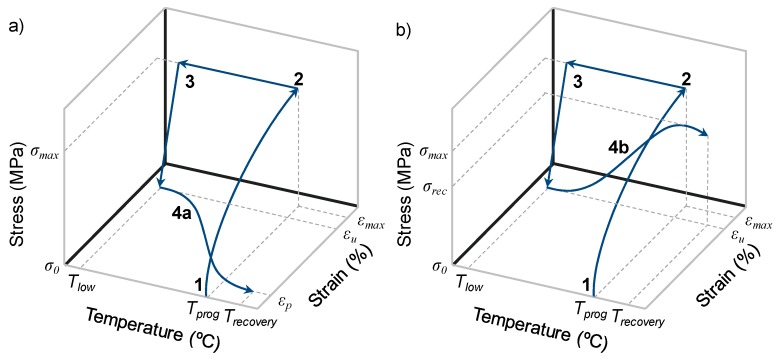
Schematic representation of the thermomechanical programming under free recovery conditions (**a**) and under fully constrained conditions, which generates a recovery stress (**b**).

The shape-memory properties were measured using the TA Instruments DMA Q800 (New Castle, Delaware, USA) in the force-controlled mode and equipped with the film-tension clamp. Dog-bone shape samples of about 20 mm × 2 mm × 0.5 mm were used in shape-memory performances. The method for creating a temporary shape and triggering the shape-memory effect is a thermomechanical procedure called programming (Figure 3). This method consists of various steps. First, the sample is heated to programming temperature *T_prog_* and deformed to a prescribed value of maximum stress *σ_max_* at 1 N/min (step 1). In this stage, the deformation of the sample is *ε_max_*. The next step is to cool the sample below the transition temperature (*T_low_*) in order to fix the temporary shape (step 2). After fixation, the stress is released at the same stress rate of 1 N/min (step 3). In this stage, the deformation of the sample is *ε_u_*. The shape-memory effect is triggered by heating the sample to a temperature above the transition temperature (step 4). The heating rate during shape recovery was 3 °C/min and the final recovery temperature *T_recovery_* was *T_g_* + 50 °C to ensure complete recovery. When the recovery process is triggered under free conditions, the amount of non-recoverable deformation at the end of programming is *ε_p_* (Figure 3a).

In order to evaluate the recovery stress, the deformation after unloading *ε_u_* was held constant while the sample was heated to the recovery temperature *T_recovery_*. In this case, the SMP generates a recovery stress, which increases as the temperature increases until a maximum value noted as *σ_rec_* (Figure 3b).

Every sample was stretched to a determined value of stress corresponding to 75% of the stress at break at *T_prog_* (*σ_max_* = 0.75·*σ_b_*) in order to perform a comparative study with the same level of load for each sample. The programming temperature was chosen as the onset of the glass transition temperature *T_g_*^*E*′^ of each sample. According to Yakacki et al. [34] a peak in the deformability of shape-memory acrylate-based polymer can be obtained at a temperature coinciding with the onset of glass transition temperature. Feldkamp and Rousseau [35] demonstrated that this phenomenon is also present in epoxy-based systems.

The most significant parameters for quantifying shape-memory properties are the shape-recovery ratio (*R_r_*) and the shape-fixity ratio (*R_f_*). The shape-recovery ratio *R_r_* (Equation (3)) quantifies the ability of the SMP to recover its original shape and was calculated as the total deformation recovered with respect to the maximum deformation reached during the programming. In order to evaluate the evolution of the recovery process as a function of temperature during the recovery stage, the *R_r_* can be reformulated as *R_r,T_* (Equation (4)) by using the deformation of the SMP at each temperature *ε_T_*. The shape-fixity ratio quantifies the ability of the SMP to fix the temporary shape. It was calculated as the deformation after the stress was released with respect to the maximum deformation (Equation (5)).

(3) $$R_{r}\left(\%\right)=\frac{\varepsilon_{max}-\varepsilon_{p}}{\varepsilon_{max}}\cdot 100$$

(4) $$R_{r,T}\left(\%\right)=\frac{\varepsilon_{max}-\varepsilon_{T}}{\varepsilon_{T}}\cdot 100$$

(5) $$R_{f}\left(\%\right)=\frac{\varepsilon_{u}}{\varepsilon_{max}}\cdot 100$$

Another interesting parameter for the evaluation of the shape-memory performance of a SMP is the shape-recovery rate (*V_r_*) [36]. This quantifies the velocity at which the permanent shape is recovered. *V_r_* was calculated as the time interval between 15% and 85% of strain recovered (Equation (6)).

(6) $$V_{r}\left(\%/min\right)=\frac{\left(\frac{\varepsilon_{rec,15\%}-\varepsilon_{rec,85\%}}{\varepsilon_{rec,15\%}}\right)\cdot 100}{\Delta t_{15\%-85\%}}$$

## 3. Results

### 3.1. Thermal Characterization

**Figure 4:**
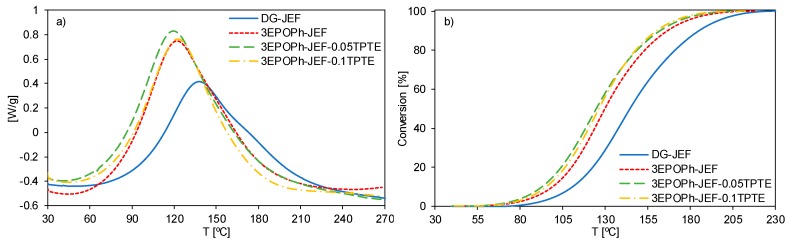
(**a**) Differential scanning calorimetry (DSC) thermograms corresponding to the dynamic curing at 10 °C/min of the formulations studied. (**b**) Conversion as a function of temperature of the formulations studied.

The curing process of the selected formulations was monitored by DSC. Figure 4 shows the DSC thermograms and Table 2 collects the most important calorimetric data obtained. As can be observed, formulations with 3EPOPh begin to react at lower temperatures compared with the formulation with DGEBA. The differences in the electronic density of the 3EPOPh molecule in comparison to DGEBA affect the reactivity of the glycidyl moiety, making the phloroglucinol derivative more reactive. The addition of TPTE does not have any significant effect on the polymerization process. The height of the peak is higher in formulations with 3EPOPh, indicating a faster curing process.

From the data in Table 2, it is possible to see that formulations with 3EPOPh show slightly lower curing enthalpy than the usual for an epoxy-amine reaction, which is described in the literature to be around 110 kJ/eq [37] and lower than the measured in the curing of DG-JEF formulation. The compact structure and high functionality of the triglycidyl phloroglucinol derivative, with three glycidyl groups linked to an only phenyl ring, could hinder the complete polymerization due to topological restrictions, reducing the enthalpy evolved. Low results of curing enthalpy were obtained as well with a triglycidyl eugenol derivative [24,26] and a tetraglycidyl bis-eugenol derivative [25] when reacted with thiols and amines.

The thermosetting material obtained from formulation 3EPOPh-JEF showed a higher glass transiton temperature than the obtained with DG-JEF. This increase is derived from the trifunctionality of 3EPOPh, which generates materials with higher crosslinking density (Table 1). However, the addition of TPTE causes a dramatical decrease in the *T_g_*s. With just a 2.4 and a 4.8 wt.% of TPTE in the composition (3EPOPh-JEF-0.05TPTE and 3EPOPh-JEF-0.1TPTE, respectively), glass transition temperatures decreased 13 °C and 27 °C with respect to the *T_g_* of 3EPOPh-JEF. Although TPTE is trifunctional such as 3EPOPh, TPTE has a more flexible structure since there is not any phenyl ring in its structure, as in the case of 3EPOPh, that introduces mobility restrictions.

**Figure 5:**
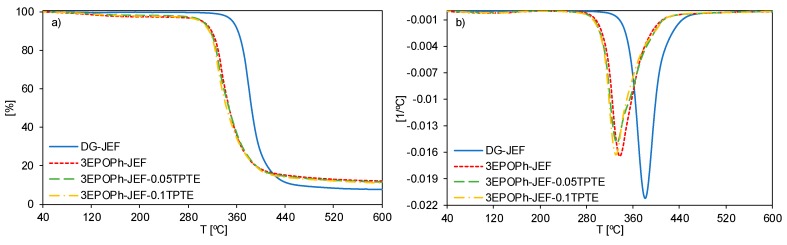
TGA (**a**) and derivative thermogravimetric curves (DTG) (**b**) under N_2_ atmosphere at 10 °C/min of the materials studied.

The materials obtained were characterized by thermogravimetry to determine their stability at high temperatures. Figure 5a shows the weight loss curves as a function of temperature in inert atmosphere and Figure 5b shows the derivatives of weight loss curves. Table 3 collects the most significant data obtained by this technique. From the temperatures of initial weight loss (*T_5%_*) and the temperatures of maximum degradation rate (*T_max_*) it can be observed that formulations with 3EPOPh have lower degradation temperatures in comparison with the prepared with DGEBA. Notwithstanding, it can be observed that the degradation rates are significantly slower when using 3EPOPh (Figure 5b) due to its higher crosslinking density that hinders the weight loss. The addition of TPTE causes a slightly decrease in the degradation temperatures. In any case, the initial degradation temperatures are always higher than 300 °C, which is more than enough for the applications for which these SMPs will be used.

The thermosets prepared with 3EPOPh have a higher charring residue because the aromatic structure of this compound rends a high C/H ratio and therefore a high carbonaceous residue is formed. These results could be positive for these materials to get a high fire retardancy.

### 3.2. Thermomechanical Characterization

**Figure 6:**
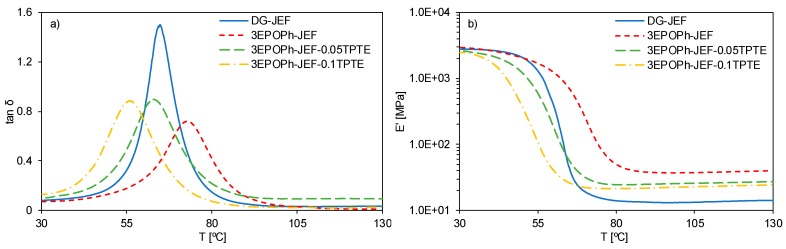
Tan δ (**a**) and *E*′ (**b**) curves of the materials prepared.

The thermomechincal characteristics of the materials obtained were determined by DMA. Figure 6 shows the DMA curves registered and Table 4 collects the most typical thermomechanical data.

The shape of the tan δ peak can be correlated with the network structure. The higher and narrower the peak of tan δ, the more homogeneous and mobile is the network structure [38]. In comparison with the formulation with DGEBA, the thermoset 3EPOPh-JEF shows a lower peak and a wider curve. The smaller size of 3EPOPh molecule and its trifunctionality, in comparison with the long and bifunctional DGEBA molecule, lead to a more heterogeneous and tighter network and to an increase in the glass transition temperature (Table 4). This material also shows a significant increase in the rubbery modulus *E*′_*r*_ in comparison with the DGEBA thermoset. According to the theory of rubber elasticity, the rubbery modulus *E*′_*r*_ is proportional to the crosslinking density [39]. Considering the values of *E*′_*r*_ (Table 4) and the estimated values of crosslinking density of Table 1, it is clear that thermoset 3EPOPh-JEF has a higher crosslinking density than DG-JEF because its trifunctionality and higher amount of crosslinking points by weight unit. A higher crosslinking density leads to a higher *T_g_*, but it also has to be considered the chemical structure of the network formed: the small and short structural unit of 3EPOPh in contrast to the longer and kinked structure of DGEBA.

The presence of TPTE in the 3EPOPh thermosets causes a significant decrease of the glass transition temperatures. Replacing part of the 3EPOPh by TPTE increases the molecular mobility between crosslinking points since TPTE has a more flexible structure. The addition of TPTE also causes a slight increase in the damping characteristics and in the height of the tan δ peak of the network because of the mobility introduced by its aliphatic nature. The presence of TPTE in the material leads to a progressive decrease in the rubbery modulus (Table 4). If one bears in mind the crosslinking density *υ_c_* assuming that all amine groups turn into crosslinks, the rubbery modulus is decreased when TPTE is added but keeping the crosslinking density constant for formulations with 3EPOPh (Table 1). Enhancing network mobility without any decrease in the crosslinking density (or even increasing it) is a common topic of study in the field of SMPs [40,41] and might benefit the shape-memory properties. 

An indicator of the shape-memory performance of a SMP is the difference between the storage modulus at glassy and rubbery regions (*E*′_*g*_/*E*′_*r*_). This ratio should be around 2 orders of magnitude if a polymer is going to present SME [5]. As can be observed in Table 4, these materials fulfil this requirement and therefore they are expected to have good shape memory characteristics.

### 3.3. Mechanical Characterization

The mechanical performance of the materials prepared were studied at room temperature (*T_room_*) and at *T_g_*^*E*′^. The values obtained are listed in Table 5. 

As can be observed, the replacement of DGEBA by 3EPOPh causes a significant increase in the stiffness and hardness of the final thermoset. At room temperature, the stress at break barely changes. However, the strain at break decreases significantly from 10.7% to 3.2%. This decrease represents a reduction of the deformability around a 70%. The microindentation hardness and the Young’s modulus increase approximately in the same magnitude. These differences in the mechanical properties are due to the trifunctionality and compactness of the aromatic 3EPOPh molecule, which increases notably the proportion of crosslinking points of the thermoset prepared from 3EPOPh.

The addition of TPTE to the 3EPOPh thermosets causes a significant decrease in the stiffness and the microindentation hardness of the final thermosets, because of the flexible structure of this aliphatic epoxy monomer. The material obtained from the 3EPOPh-JEF-0.05TPTE formulation, with just a 2.4 wt.% of TPTE, shows approximately the same mechanical properties at room temperature than the obtained from the formulation DG-JEF. A higher amount of TPTE (4.8 wt.% in the formulation 3EPOPh-JEF-0.1TPTE) leads to a further decrease in the rigidity and microhardness. The flexible structure of TPTE enhances molecular mobility and allows materials to increase their deformability, but without any significant change in their stress capability.

When the mechanical characteristics are evaluated at *T_g_*^*E*′^, the trend is similar to the observed at room temperature. It must be taking into account that *T_g_*^*E*′^ is a temperature close to the *T_g_* and therefore the mateials are partially relaxed at this temperature. The thermoset 3EPOPh-JEF showed higher stress at break and significantly lower strain at break than the obtained with formulation with DGEBA. When TPTE is added, the stress-strain capability of the materials is enhanced in comparison with formulation 3EPOPh-JEF, but the deformability remains well below the value of 63% shown by the thermoset obtained from DGEBA. Remarkable values are obtained with formulation 3EPOPh-JEF-0.05TPTE, which shows high values of stress at break, almost 12 MPa, barely seen in these kind of SMPs at elevated temperatures [36].

### 3.4. Shape-Memory Properties

**Figure 7:**
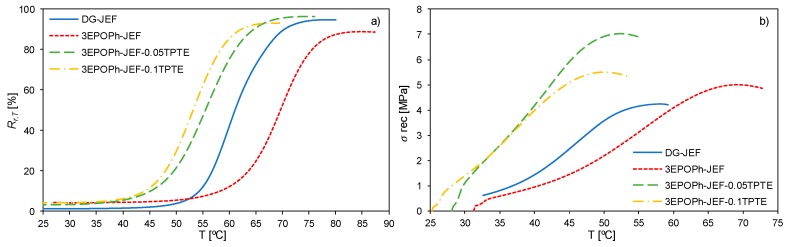
(**a**) Shape-recovery ratio as a function of temperature during the recovery stage under free conditions of all formulations. (**b**) Recovery stress generation as a function of temperature during the recovery stage under constrained conditions of all formulations.

In order to perform a comparative study, every sample was stretched during shape-memory programming to a prescribed maximum stress (*σ_max_*) corresponding to the 75% of the stress at break. The prescribed maximum stress *σ_max_* and the corresponding maximum deformation reached *ε_m_* of each sample are listed in Table 5. Figure 7 shows the recovery stage of the programming (step 4 in Figure 3) of each sample with both methods: under free conditions (Figure 7a) and under constrained conditions (Figure 7b). When the recovery stage is performed under free conditions, the SMP progressively recovers its original shape while the temperature is increasing. This can be observed as an increase of shape-recovery ratio *R_r,T_* (Equation (3)) as the temperature increases. In the case of the recovery stage performed under constrained conditions, the deformation after unloading the maximum stress applied during the programming *ε_u_* is held constant, so that the SMP cannot recover its original shape when the temperature increases. Instead, the SMP exercises a recovery stress, which increases with temperature until a maximum.

The shape-memory parameters are listed in Table 6. As can be observed, the shape-memory performance of the thermoset DG-JEF was excellent. It showed almost complete shape-recovery and shape-fixation as well as very high shape-recovery rate and recovery stress. Systems formed by a DGEBA and a Jeffamine^®^ of different molecular weights are well studied and their shape-memory properties reported in several research papers [40,41,42]. Replacing DGEBA with 3EPOPh leads to a slight decrease in the shape-memory properties. However, when TPTE is added to the formulation the shape-memory properties are enhanced (in comparison with 3EPOPh-JEF), reaching a level of performance as good as formulation DG-JEF. 

Shape-memory properties depend on a combination of factors related to network structure and thermomechanical properties. During the programming step, the material can be easily deformed due to the increase in chain mobility. When the applied external stress is released at low temperature, there is a small elastic recovery coming from the immediate relaxation of local deformations. However, the lower mobility of the network structure prevents chain rearrangement and holds the temporary deformation. Replacing DGEBA with 3EPOPh just causes a slight decrease in the *R_f_* but the shape fixation is maintained higher than 95% in all cases.

Once the temperature is high enough, the network structure gains enough mobility and the internal stresses are eventually relaxed. Thus, the SMP is able to recover its original shape. On the one hand, the SMP must have a certain degree of crosslinking density, which is the responsible for exercising the driving recovery force during the recovery stage, but the shape-memory properties can also be rationalized in terms of viscoelastic properties [36,43,44]. Replacing DGEBA with 3EPOPh causes a decrease in the *R_r_*. Although the higher crosslinking density of 3EPOPh, network structure of samples prepared with 3EPOPh have more heterogeneous structure and more restricted mobility. From the DMA analysis shown in Figure 6 and Table 4, it can be seen that formulations with 3EPOPh have broader network relaxation, so the shape-recovery process will be more hindered than in the case of the more mobile and homogeneous thermoset prepared with DGEBA.

As observed in Figure 6 and Table 4, a small wt.% of TPTE has a dramatic effect on the viscoelastic properties and so does it in the shape-memory properties. Formulation 3EPOPh-JEF-0.05TPTE, with just a 2.4 wt.% of TPTE, shows a significant enhancement in the *R_r_*, even higher than the value with DGEBA. However, a higher amount of TPTE could be harmful since the *R_r_* slightly decrease in the case of formulation 3EPOPh-JEF-0.1TPTE. By introducing the appropriate amount of a rubber-like modifier, like TPTE, the molecular mobility and network homogeneity is enhanced without affecting the driving recovery forces.

In the case of the shape-recovery rate *V_r_*, network mobility acquires more relevance as *V_r_* is increased with the wt.% of TPTE. The SMP will take less time to recover its original shape in a more mobile network. Therefore, it can be assumed that network relaxation dynamics rather than the theoretical crosslinking density is the key parameter governing the recovery process [36,43,44].

The recovery stress generation during the recovery stage under constrained conditions comes from a combination of the maximum stress applied during the programming of the temporary shape, the driving recovery forces exercised by the crosslinked network and, again, network homogeneity. On one side, the higher the stress at break *σ_b_*, the higher maximum stress during programming *σ_max_* the SMP can endure; and the higher *σ_max_*, the higher *σ_rec_* during constrained shape-recovery stage. In addition, some studies have concluded that the crosslinking density is the governing factor in recovery stress generation due to the larger driving entropy recovery forces [45,46]. However, in previous papers, our research group stated the importance of the molecular mobility and homogeneity [47,48].

The values of *σ_rec_* shown in Table 6 follow the trend explained before (the higher *σ_max_*, the higher *σ_rec_*). It mus be noted the remarkable value of 7 MPa of formulation 3EPOPh-JEF-0.05TPTE, which has been rarely seen in the literature [47,49,50]. Taking into account the different values of *σ_max_* (*σ_max_* = 0.75·*σ_b_*) applied to the different samples, the recovery stress to maximum stress ratio *σ_rec_*/*σ_max_* were calculated in order to rationalize the recovery stress generation in terms of viscoelastic properties. In the case of the thermoset DG-JEF, the recovery stress generated was almost equal to the stress reached during the deformation step *σ_max_* (*σ_rec_*/*σ_max_* = 94%). When DGEBA is replaced with 3EPOPh, the *σ_rec_*/*σ_max_* ratio decreased to ~84%, and with TPTE it decreased to ~80% and 75% with 2.4 wt.% and 4.8 wt.% respectively. Again, the fact that the network homogeneity is reduced in materials with 3EPOPh, in comparison with materials with DGEBA, explains this decrease despite the fact that the crosslinking density is increased (Table 1). The *σ_rec_*/*σ_max_* ratio decreases while increasing the content of TPTE, since the driving recovery forces are decreased in a network with higher presence of more viscous-like segments between crosslinking points (coming from TPTE) than in a bulk network formed by more elastic-like segments from 3EPOPh.

## 4. Conclusions

A series of bio-based thermosetting shape-memory polymers were synthesized using triglycidyl phloroglucinol previously synthesized and the commercial trimethylolpropane triglycidyl ether as epoxy monomers and a polyetheramine as crosslinking agent. The materials obtained were compared with a standard shape-memory thermoset prepared with DGEBA and were characterized by means of DSC, TGA, DMA, stress-strain tests, and microindentation. Shape-memory properties were evaluated under free and constrained conditions.

The DSC study revealed that when replacing DGEBA with 3EPOPh, the polymerization of the formulations began at lower temperatures and showed faster reaction rates. The curing reaction of formulations with 3EPOPh showed lower enthalpy by epoxy equivalent than the formulation with DGEBA due to topological restrictions. The material obtained from formulation 3EPOPh-JEF showed a higher glass transiton temperature than the obtained with DGEBA, due to the trifunctionality and rigid structure of 3EPOPh. The addition of TPTE caused a dramatically decrease of the *T_g_*s. Materials prepared from 3EPOPh showed good thermal stability, with degradation temperatures higher than 300 °C.

Replacing DGEBA with 3EPOPh led to a tighter network with higher crosslinking density and glass transition temperatures. The addition of TPTE caused an increase in the molecular mobility and a decrease of the glass transition temperatures. The SMPs prepared from 3EPOPh showed good mechanical properties as well as a good shape-memory performance, especially in combination with small quantities of TPTE. They showed almost complete shape-recovery and shape-fixation, fast shape-recovery rate and remarkable values of recovery stress up to 7 MPa.

The results obtained in this study allow us to conclude that the triglycidyl phloroglucinol derivative of eugenol 3EPOPh is a safe and environmentally friendly alternative to DGEBA for thermosetting shape-memory polymers.

## Figures and Tables

**Table 1:** Percentages in weight of epoxy monomers and crosslinking agent used in the preparation of the formulations.

Sample	DGEBA [wt. %]	3EPOPh [wt. %]	TPTE [wt. %]	JEF [wt. %]	υ_c_^1^ [mol/g]	υ_e_^2^ [mol/g]
DG-JEF	62.9	0	0	37.1	0.0017	0.0014
3EPOPh-JEF	0	47.7	0	52.3	0.0024	0.0039
3EPOPh-JEF-0.05TPTE	0	45.3	2.4	52.3	0.0024	0.0027
3EPOPh-JEF-0.1TPTE	0	43.0	4.8	52.2	0.0024	0.0024

^1^ Crosslinking density calculated assuming that all amine groups turn into crosslinks. ^2^ Crosslinking density calculated using Equation (1).

**Table 2:** Calorimetric data of the curing process of the formulations studied.

Sample	*T_peak_* [°C]	*ΔH* [J/g] ^1^	*ΔH* [kJ/eq] ^2^	*T_g_* [°C] ^3^
DG-JEF	140	386	112	56
3EPOPh-JEF	124	488	100	61
3EPOPh-JEF-0.05TPTE	121	504	105	48
3EPOPh-JEF-0.1TPTE	125	429	88	34

^1^ Enthalpy by gram of sample in a dynamic curing. ^2^ Enthalpy by equivalent epoxy of sample in a dynamic curing. ^3^ Glass transition temperature of the material after thermal curing.

**Table 3:** Thermogravimetric data of the materials prepared.

Sample	T_5%_ [°C] ^1^	T_max_ [°C] ^2^	Char Residue [%]
DG-JEF	354	381	7.6
3EPOPh-JEF	304	338	12.0
3EPOPh-JEF-0.05TPTE	303	332	11.5
3EPOPh-JEF-0.1TPTE	301	331	11.1

^1^ Temperature of 5% of weight loss in N_2_ atmosphere. ^2^ Temperature of the maximum rate of degradation in N_2_ atmosphere.

**Table 4:** Thermomechanical data of the materials studied obtained by DMA.

Sample	*T_g_*^1^ [°C]	*T_g_*^*E*′^^2^ [°C]	tan δ Peak	FWHM ^3^ [°C]	*E*′_*g*_^4^ [MPa]	*E*′_*r*_^5^ [MPa]	*E*′_*g*_/*E*′_*r*_
DG-JEF	65	57	1.5	10	2770	14	198
3EPOPh-JEF	73	60	0.7	17	2942	39	75
3EPOPh-JEF-0.05TPTE	63	49	0.9	18	2584	26	99
3EPOPh-JEF-0.1TPTE	56	42	0.9	18	2460	23	107

^1^ Measured as the peak of tan *δ.* ^2^ Measured as the peak of E″. ^3^ Full Width at Half Maximum. ^4^ Measured at room temperature. ^5^ Measured at T_g_+50 °C.

**Table 5:** Mechanical properties of the materials studied at *T_ro__om_* and *T_g_*^*E*′^. In the case of *σ_b_*, *ε_b_*, and *E* the mean value of three different samples tested is shown. Coefficients of variations are less than 7% for stress and strain results, and less than 5% for the Young’s moduli.

Sample	*T_room_*				*T_g_* ^*E*′^			
	*σ_b_*^1^ [MPa]	*ε_b_*^2^ [%]	Micro-Indendation [HV]	*E*^3^ [MPa]	*σ_b_*^1^ [MPa]	*ε_b_*^2^ [%]	*σ_max_*^4^ [MPa]	*ε_max_*^5^ [%]
DG-JEF	30.0	10.7	5.4 ± 0.4	1318	6.0	63.3	4.5	46.8
3EPOPh-JEF	28.0	3.2	8.7 ± 0.2	2222	8.2	27.1	6.1	16.0
3EPOPh-JEF-0.05TPTE	27.7	10.4	5.8 ± 0.9	1592	11.7	36.8	8.7	25.5
3EPOPh-JEF-0.1TPTE	27.0	29.8	4.1 ± 0.2	1372	9.7	37.9	7.3	27.0

^1^ Stress at break. ^2^ Strain at break. ^3^ Young’s modulus determined under flexural conditions. ^4^ Prescribed maximum stress applied corresponding to the 75% of *σ_b_* at *T_g_*^*E*′^. ^5^ Maximum strain reached during shape-memory programming.

**Table 6:** Shape-memory properties of the materials studied.

Sample	*R_r_* [%]	*R_f_* [%]	*V_r_* [%/min]	*σ_rec_*^1^ [MPa]	*σ_rec_*/*σ_max_*^2^ [%]
DG-JEF	94.6	98.8	19.8	4.3	94.2
3EPOPh-JEF	88.7	95.7	15.3	5.0	83.7
3EPOPh-JEF-0.05TPTE	96.2	96.6	16.8	7.0	80.8
3EPOPh-JEF-0.1TPTE	93.1	95.6	18.4	5.5	75.2

^1^ Maximum recovery stress developed during the recovery stage under constrained conditions. ^2^ Maximum recovery stress to maximum stress applied ratio.
